# Multi‐layered inhibition of *Streptomyces* development: BldO is a dedicated repressor of *whiB*

**DOI:** 10.1111/mmi.13663

**Published:** 2017-03-27

**Authors:** Matthew J. Bush, Govind Chandra, Kim C. Findlay, Mark J. Buttner

**Affiliations:** ^1^ Department of Molecular MicrobiologyJohn Innes Centre, Norwich Research ParkNorwichNR4 7UHUK; ^2^ Department of Cell and Developmental BiologyJohn Innes Centre, Norwich Research ParkNorwichNR4 7UHUK

## Abstract

BldD‐(c‐di‐GMP) sits on top of the regulatory network that controls differentiation in *Streptomyces*, repressing a large regulon of developmental genes when the bacteria are growing vegetatively. In this way, BldD functions as an inhibitor that blocks the initiation of sporulation. Here, we report the identification and characterisation of BldO, an additional developmental repressor that acts to sustain vegetative growth and prevent entry into sporulation. However, unlike the pleiotropic regulator BldD, we show that BldO functions as the dedicated repressor of a single key target gene, *whiB*, and that deletion of *bldO* or constitutive expression of *whiB* is sufficient to induce precocious hypersporulation.

## Introduction

Streptomycetes are a genus of Gram‐positive bacteria abundant in soil. Within this habitat, they are found in two distinct forms: as a multicellular mycelium and as dormant unicellular spores (Flärdh and Buttner, 2009; McCormick, 2009; McCormick and Flärdh, 2012; Jakimowicz and van Wezel, 2012; Bush *et al*., 2015). Under favourable nutrient conditions, streptomycetes grow by tip extension and branching as filamentous hyphae to form a vegetative mycelium that facilitates nutrient scavenging (Flärdh *et al*., 2012). This mycelium is multicellular but delimited by occasional hyphal cross‐walls. In response to nutrient depletion, streptomycetes initiate reproductive growth, ultimately forming unicellular spores that can survive adverse conditions and promote dispersal. Differentiation begins with the formation of reproductive structures called aerial hyphae that grow away from the surface of the vegetative mycelium into the air, giving the colonies a characteristic fuzzy appearance. Subsequently, each aerial hypha arrests tip growth and initiates a massive cell division event involving the synchronous formation of dozens of sporulation septa that divide the multigenomic tip cell into a long chain of unigenomic prespore compartments. These compartments further differentiate before finally being released as thick‐walled, dormant spores.

The key regulators that orchestrate this developmental pathway fall into two classes (Flärdh and Buttner, 2009; McCormick and Flärdh, 2012; Bush *et al*., 2015). Bld (Bald) regulators are required for the formation of the hair‐like reproductive structures, and so mutations in *bld* loci result in a ‘bald’ phenotype. Whi (white) regulators are required for the differentiation of aerial hyphae into mature spores, and mutations in *whi* loci therefore prevent synthesis of the polyketide spore pigment that normally gives mature colonies their characteristic colour. Two of the developmental regulators particularly relevant to this article are BldD and WhiB.

BldD sits at the top of the regulatory cascade controlling development, serving to repress expression of a large regulon of sporulation genes during vegetative growth (Elliot *et al*., 2001; den Hengst *et al*., 2010). An important recent insight into the regulation of *Streptomyces* development is that cyclic‐di‐GMP (c‐di‐GMP) signals through the master regulator BldD to control entry into sporulation. c‐di‐GMP mediates the dimerization of two BldD protomers, leading to DNA binding (Tschowri *et al*., 2014; Bush *et al*., 2015). In this way, c‐di‐GMP drives repression of the BldD regulon, extending vegetative growth and inhibiting the hypha‐to‐spore transition. Loss of BldD removes this developmental ‘brake’, and so *bldD* mutants are bald not because they fail to initiate development, but because the entire biomass sporulates precociously, bypassing the formation of aerial hyphae (Tschowri *et al*., 2014; Bush *et al*., 2015). Because it is a complex of c‐di‐GMP bound to BldD that represses the expression of sporulation genes during vegetative growth, not BldD alone, precocious hypersporulation can also be engineered by depleting intracellular c‐di‐GMP levels (Tschowri *et al*., 2014; Bush *et al*., 2015).

*whiB* is one of the key targets repressed by BldD. *whiB* and *whiA* mutants fail to halt aerial growth, to initiate the synchronous septation event or to partition their chromosomes. Instead, the cells keep growing, producing long aerial hyphae devoid of sporulation septa and containing uncondensed DNA (Flärdh *et al*., 1999; Aínsa *et al*., 2000; Bush *et al*., 2013, 2016). These identical phenotypes arise because WhiA and WhiB are transcription factors that function cooperatively to co‐control expression of a common set of WhiAB target genes (Bush *et al*., 2013, 2016). *whiA* is one of the few genes of the core transcriptional regulatory cascade that is not directly regulated by BldD (den Hengst *et al*., 2010; Bush *et al*., 2013, 2015). Instead, WhiA is present throughout the life cycle and WhiAB function seems to be controlled through WhiB (Bush *et al*., 2013, 2016, 2015).

Our understanding of developmental regulation in *Streptomyces* derives from three model species. Historically, the most significant model, and the one in which most of the developmental regulators were identified, has been *Streptomyces coelicolor*, but important insights have also been derived from analysis of the streptomycin producer, *Streptomyces griseus*. Recently, however, the chloramphenicol producer *S. venezuelae* has emerged as a new model species with several attractive characteristics.

Unlike *S. coelicolor*, which only sporulates on plates, *S. venezuelae* also sporulates in liquid media (Glazebrook *et al*., 1990; Bush *et al*., 2015). Because of this ability, the adoption of *S. venezuelae* has greatly facilitated the application of global ‘omics’ approaches to dissect the regulatory network that controls differentiation (Bibb *et al*., 2012; Bush *et al*., 2013, 2016, 2015; Al‐Bassam *et al*., 2014; Tschowri *et al*., 2014). Equally, sporulation in liquid has allowed time‐lapse live‐cell imaging of sporulation using a microfluidic device (Donczew *et al*., 2016; Schlimpert *et al*., 2016). As a consequence, it is now possible to monitor the subcellular localization of fluorescently tagged proteins in movies covering the complete spore‐to‐spore life cycle (Donczew *et al*., 2016; Schlimpert *et al*., 2016).

Here, using *S. venezuelae*, we characterize BldO, a newly identified developmental repressor that, like BldD, acts to sustain vegetative growth and prevent entry into sporulation. However, unlike the global regulator BldD, we show that BldO functions as the repressor of a single key target gene, *whiB*, and that deletion of *bldO* or constitutive expression of *whiB* is sufficient to induce precocious hypersporulation.

## Results

### Deletion of *bldO* causes precocious hypersporulation

**Figure 1:**
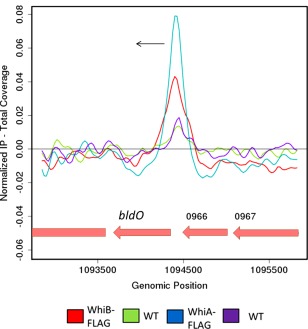
ChIP‐seq data showing that *bldO* is a WhiAB target. Colour‐coding of the ChIP samples is as follows: 3xFLAG‐[Gly_4_Ser]_3_‐WhiB strain (WhiB‐FLAG, red), corresponding *S. venezuelae* wild‐type anti‐FLAG negative control (WT, green), 3xFLAG‐[Gly_4_Ser]_3_‐WhiA strain (WhiA‐FLAG, blue) and corresponding *S. venezuelae* wild‐type anti‐FLAG negative control (WT, purple). The plot spans approximately 3 kb of DNA sequence. Genes running right to left are shown in red. The black arrow indicates the gene subject to WhiA and WhiB regulation (*bldO*). These data are derived from the ChIP‐seq datasets described in Bush *et al*., *mBio*, 2016, 7, e00523‐16 (with permission), but *sven0965* (*bldO*) was not specifically discussed in that paper.

**Figure 2:**
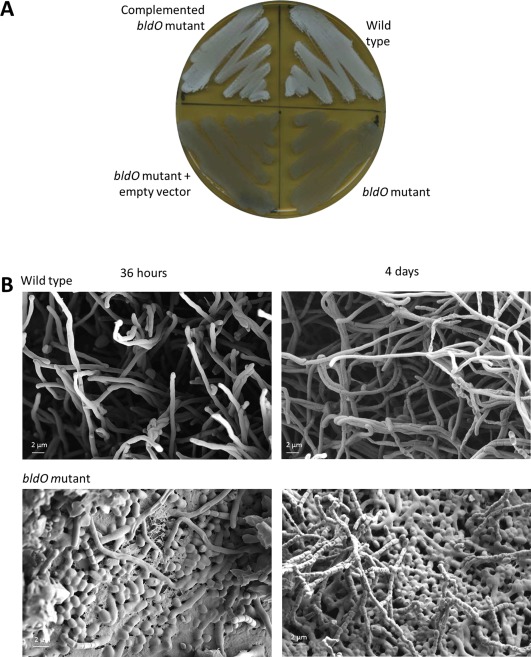
Deletion of *bldO* causes precocious hypersporulation on solid medium. A. The phenotypes of wild‐type *S.venezuelae*, the *bldO* mutant, the *bldO* mutant carrying the empty vector, and the complemented *bldO* mutant, photographed after four days of growth on DNA medium. B. Scanning electron micrographs showing the precocious hypersporulation phenotype of the *bldO* mutant after 36 h and 4 days of growth on DNA medium.

We constructed null mutants for a number of genes that we previously identified as direct regulatory targets of WhiAB (Bush *et al*., 2013, 2016). One of these targets was *sven0965* (here designated *bldO*) (Fig. 1). *bldO* is universally conserved in all sequenced *Streptomyces* genomes, and encodes a putative transcription factor. The resulting mutant was bald (Fig. 2A), making small, soft, friable colonies that were almost devoid of aerial hyphae. Scanning electron microscopy (SEM) showed that despite the lack of an aerial mycelium, deletion of *bldO* in fact promoted sporulation, but the colonies appeared bald to the naked eye because aerial mycelium formation had been bypassed (Fig. 2B). The biomass of even young colonies consisted almost entirely of spores (Fig. 2B), suggesting that loss of BldO function accelerated entry into development, potentially consistent with the small size of the mutant colonies.

The developmental phenotype was also striking in liquid culture. Wild‐type *S. venezuelae* typically begins to differentiate after ∼16 h in liquid, when the small mycelial clumps seen during vegetative growth start to fragment and spores begin to form. In contrast, at the equivalent time point, the *bldO* mutant culture consisted almost entirely of spores (Supporting Information Fig. S1). Importantly, on agar and in liquid culture, the *bldO* mutant phenotype was fully complemented by introducing a single copy of the *bldO* gene under the control of its native promoter, expressed *in trans* from the ΦBT1 integration site (Fig. 2A and Supporting Information Fig. S1).

**Figure 3:**
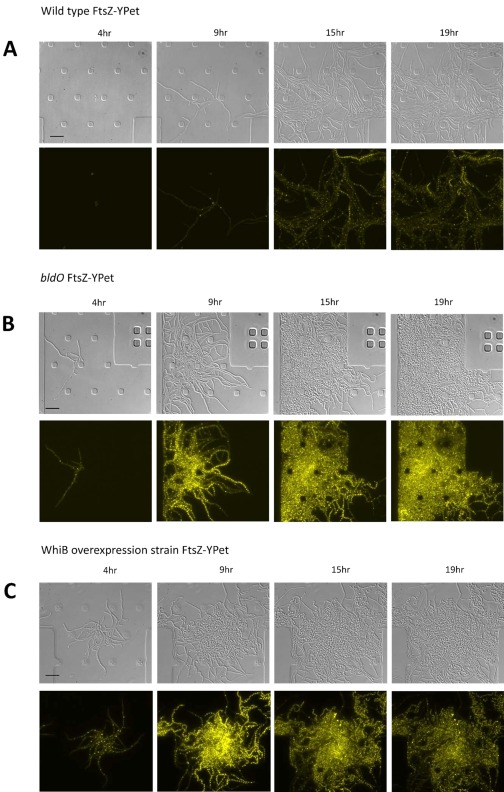
Deletion of *bldO* or overexpression of WhiB causes precocious hypersporulation in liquid medium. Time‐lapse images (4, 9, 15 and 19 h) of (A) wild‐type *S. venezuelae*, (B) the *bldO* mutant and (C) wild‐type *S. venezuelae* constitutively expressing *whiB* from the ermE* promoter, grown in DNB, in the microfluidic system. All three strains carry the same FtsZ‐YPet translational fusion expressed from the native *ftsZ* promoter, and both the DIC (upper) and fluorescence (lower) images are shown. For the corresponding movies, please see Supporting Information Movies S1A/B, S2A/B and S6A/B. Scale bars = 10 µm.

We have recently developed fluorescence time‐lapse imaging of the complete *S. venezuelae* life cycle using a microfluidic device (Schlimpert *et al*., 2016). We used this system to further analyse development of the *bldO* mutant, comparing it in parallel with wild‐type *S. venezuelae* and the complemented *bldO* strain (Fig. 3A and B, Supporting Information Movie S1A/S1B, Movie S2A/S2B and Movie S3A/S3B). For these experiments, a fluorescent FtsZ‐YPet fusion was introduced into the strains to help track the two distinct kinds of cell division that occur in *Streptomyces* (Bush *et al*., 2015). In the time‐lapse images, the scattered FtsZ rings (Z rings) seen during early growth mark the formation of so‐called vegetative cross‐walls, which divide the hyphae into long multigenomic compartments but do not lead to constriction or cell‐cell separation (Fig. 3A, second panel and Supporting Information Movie S1). In contrast, in each reproductive hypha, very bright ladders of Z rings are synchronously laid down at ∼1.3 µm intervals, leading to the formation of dozens of sporulation septa (Fig. 3A, third and fourth panels and Supporting Information Movie S1). Unlike vegetative cross‐wall formation, sporulation septation leads to constriction, cell‐cell separation and, ultimately, the release of mature, unigenomic spores. Time‐lapse imaging showed that the period of vegetative growth in the *bldO* mutant was considerably shorter than in the wild type (Fig. 3B and Supporting Information Movie S2A/B). Strikingly, in the *bldO* mutant there was a dramatic increase in the FtsZ‐YPet signal and the abundant appearance of sporulation septa after only 9 h (Fig. 3B and Supporting Information Movie S2A/B). Further, essentially the entire biomass sporulated, and the process of development was complete in just 15 h (Fig. 3B and Supporting Information Movie S2A/B). In contrast, in the wild type, the first FtsZ ladders were not observed until 14 h and many hyphae had yet to differentiate even after 24 h (Fig. 3A and Supporting Information Movie S1A/B). Importantly, the normal control and timing of sporulation septation was restored by complementation of the *bldO* mutant *in trans* (Supporting Information Movie S3A/B). We conclude that, like BldD, BldO is required to maintain a sustained period of vegetative growth prior to sporulation septation, and so precocious hypersporulation occurs when *bldO* is deleted.

### BldO levels peak at the onset of sporulation

**Figure 4:**
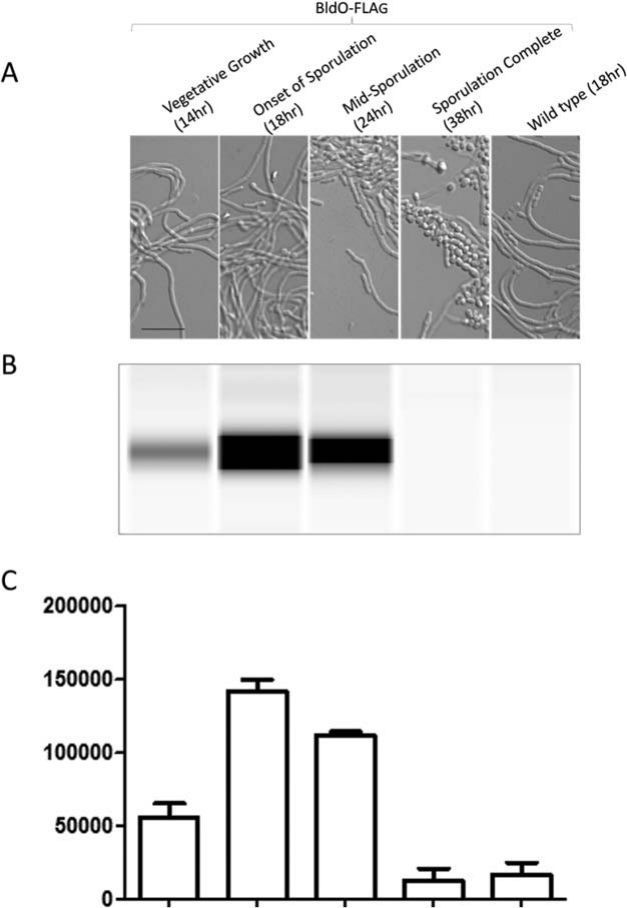
Automated Western blot analysis of the C‐terminally FLAG‐tagged version of BldO, expressed *in trans* from the native promoter in the *bldO* null mutant background. Equal amounts (2.5 µg) of total protein were loaded for each sample and BldO‐FLAG was detected with anti‐FLAG antibody using the quantitative ‘Wes’ capillary electrophoresis and blotting system (ProteinSimple – San Jose, CA; see Supplementary Material). Wild‐type *S. venezuelae* expressing non‐FLAG‐tagged BldO was used as a negative control. Both the BldO FLAG‐tagged strain and wild type were grown in DNB medium. Top: DIC images of the culture at each time point. Middle: virtual Western blot. Bottom: quantitation of BldO levels (area under each peak; arbitrary units). All experimental samples were analysed in triplicate and the mean value and its Standard Error are shown for each sample.

We constructed strains that lacked *bldO* at the native locus but instead carried a triple FLAG‐tagged version of *bldO* under the control of its native promoter at the ΦBT1 integration site. Both N‐terminally and C‐terminally triple FLAG‐tagged versions of *bldO* complemented the *bldO* null mutant, restoring normal development on agar (Supporting Information Fig. S1A) and in liquid culture (Supporting Information Fig. S1B), and the C‐terminal fusion was used for further experiments. Western blotting using anti‐FLAG antibody showed that BldO was at its highest level at the onset of sporulation, with lower levels during vegetative growth, and that BldO was undetectable when sporulation was complete. Wild‐type *S. venezuelae* was used as a negative control (Fig. 4).

**Figure 5:**
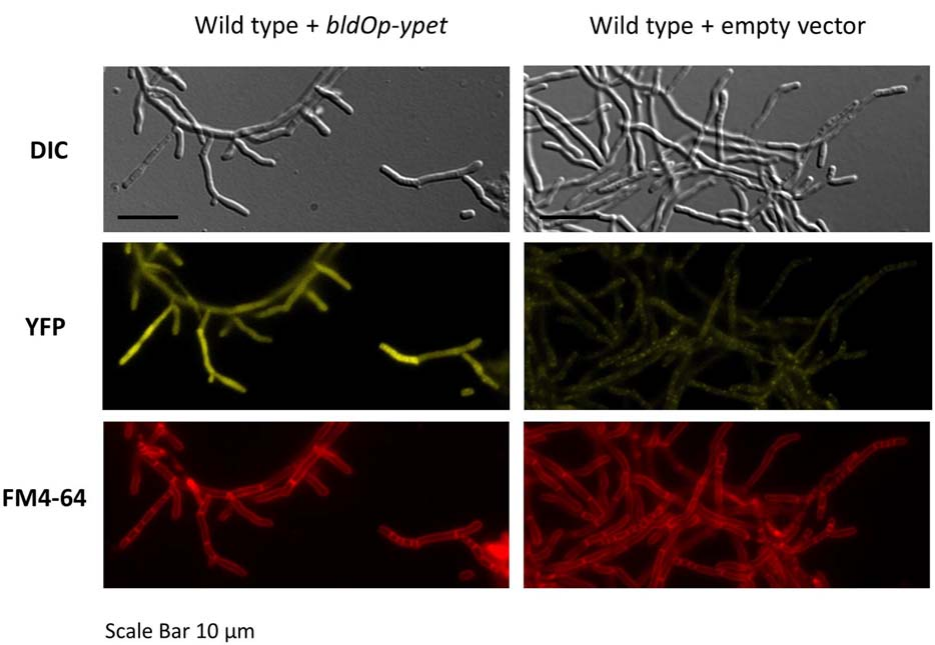
Spatial localisation of *bldO* transcription. Fluorescence images of wild‐type *S. venezuelae* carrying the *bldOp‐ypet* transcriptional fusion or the empty *ypet* reporter vector. Strains were imaged on coverslips after 18 h of growth in DNB, following the addition of FM4‐64 membrane dye. For the corresponding movies of the same strains, grown in the microfluidic system, please see Supporting Information Movies S4A/B and S5A/B.

To examine the spatial pattern of *bldO* expression, we constructed a *bldO* transcriptional reporter. We mapped the transcription start site of the *bldO* promoter (*bldOp*) by 5′ RACE and found it to lie 29 nucleotides upstream of the annotated *bldO* start codon (Supporting Information Fig. S3). With this information in hand, we made a *bldOp‐ypet* transcriptional fusion, introduced it into wild‐type *S. venezuelae*, and used the time‐lapse imaging system to determine when and where *bldO* was expressed, comparing it with the empty *ypet* reporter vector as a control (Fig. 5 and Supporting Information Movie S4A/B and Movie S5A/B). Consistent with the Western blotting data, the *bldO* promoter was upregulated in sporogenic hyphae, just prior to the formation of sporulation septa, with weaker expression in vegetative cells.

### BldO functions to repress *whiB*

**Figure 6:**
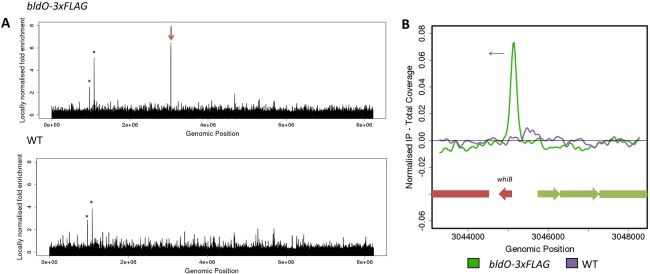
BldO has a single target, *whiB*. A. Genome‐wide distribution of BldO binding sites identified by ChIP‐seq analysis using M2 anti‐FLAG antibody, conducted at the onset of sporulation on the *bldO* null mutant complemented *in trans* with a functional *bldO‐3xFLAG* allele expressed under the control of the native promoter from the ΦBT1 integration site. Strains were grown in DNB medium. The peak upstream of *whiB* is marked by the red arrow. Peaks also seen in the negative control (wild‐type *S. venezuelae*), such as those indicated by asterisks, were excluded from further analysis. B. Close‐up of a ∼5‐kb region around *whiB*. Colour‐coding of the ChIP samples is as follows: the onset of sporulation (*bldO‐3xFLAG*, green), *S. venezuelae* wild‐type anti‐FLAG negative control (WT, purple). Genes running left to right are shown in green, and genes running right to left are shown in red. The black arrow indicates the gene (*whiB*) subject to BldO regulation.

BldO is predicted to be a DNA‐binding protein of the MerR family, and so we sought to identify the genomic positions to which BldO binds *in vivo* by chromatin immunoprecipitation‐sequencing (ChIP‐seq), using anti‐FLAG antibody to immunoprecipitate the functional C‐terminal FLAG‐tagged version of BldO. Based on the Western blotting data, we performed ChIP‐seq at the onset of sporulation when BldO levels were maximal, and used wild‐type *S. venezuelae* as a negative control to eliminate any signals that might arise from cross‐reaction of the anti‐FLAG antibody with other DNA‐binding proteins. Using *P* < 10^−4^ as the threshold for significance, only one peak was identified, located immediately upstream of *whiB* (Fig. 6A and B), suggesting that BldO regulates a single target.

**Figure 7:**
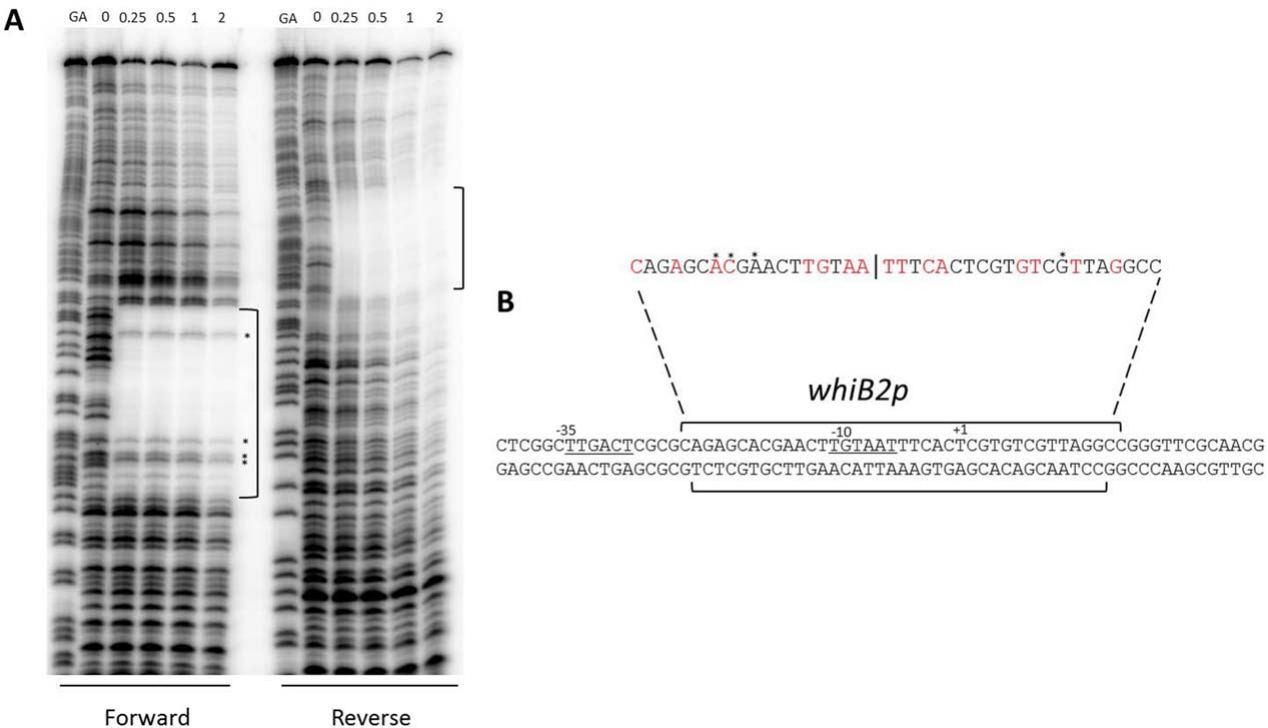
BldO binds to a sequence overlapping the transcriptional start site and the −10 region of the developmentally induced *whiBp2* promoter. A. DNase I footprinting analysis of BldO bound to radiolabelled probes derived from the (forward) and (reverse) sequence upstream of *whiB*. 5′ end‐labelled probes were incubated with increasing concentrations of BldO (indicated in µM above the lanes) and subjected to DNase I footprinting analysis. The footprints are flanked by Maxam and Gilbert sequence ladders (GA) and the black brackets indicate the positions of the BldO‐protected regions. Asterisks mark phosphodiester bonds that are not protected from cleavage. B. Summary of the DNase I footprinting results, showing the protected sequences on the forward and reverse strands (black brackets) relative to the transcriptional start site (+1) and the −10 and −35 sequences (underlined). The sequence of the protected region is also shown above with the hyphenated inverted repeat highlighted in red. Asterisks mark phosphodiester bonds that are not protected from cleavage.

We determined the exact position of the BldO binding site upstream of *whiB* using DNase I footprinting (Fig. 7A). N‐terminally his‐tagged BldO protected a ∼40 nucleotide region centred on a hyphenated inverted repeat (TGxAATTxCA) (Fig. 7B). This region overlaps the transcription start site and −10 region of the developmentally induced *whiB2p* promoter, mapped in both *S. coelicolor* (Soliveri *et al*., 1992) and in *S. venezuelae* (unpublished), suggesting that BldO might act as a repressor.

**Figure 8:**
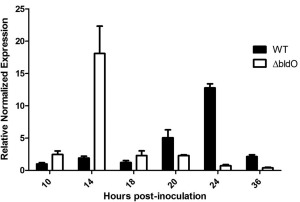
BldO functions to repress *whiB* transcription. *whiB* mRNA abundance determined by qRT‐PCR in the wild type (black bars) and the *bldO* mutant (white bars) throughout development. Strains were grown in DNB medium. Expression values were calculated relative to the accumulation of the constitutively expressed *hrdB* reference mRNA and normalised to the wild‐type value at 10 h.

To determine the regulatory effect of BldO binding to the developmentally induced *whiB2p* promoter, we used qRT‐PCR to examine the level and timing of *whiB* expression in the wild type and in the *bldO* mutant. The data showed that *whiB* expression peaks at 14 hours in the *bldO* mutant, 10 hours earlier than in the wild type (Fig. 8). Taken together, these data show that BldO functions as a dedicated repressor of *whiB* transcription.

If BldO serves only to repress expression of *whiB*, then a *bldO whiB* double mutant should have the same phenotype as a *whiB* single mutant. To test this idea, we constructed a *bldO whiB* double mutant and indeed found that it formed a normal aerial mycelium but failed to initiate sporulation septation, a phenotype identical to that of the *whiB* single mutant (Supporting Information Fig. S2).

### Overexpression of *whiB* also causes precocious hypersporulation

If the sole function of BldO is to repress expression of *whiB* during vegetative growth, then early expression of *whiB* might mimic the effect of deleting *bldO*. Accordingly, we expressed w*hiB* constitutively using the ermE* promoter in the wild‐type background and used time‐lapse imaging to characterise the resulting strain. The vegetative phase of growth of the *whiB* overexpression strain was short, with Z ladders forming early and abundantly across the entire culture (Fig. 3C and Supporting Information Movie S6A/B). When this strain was compared in parallel with the wild type (Fig. 3A and Supporting Information Movie S1A/B) and the *bldO* deletion mutant (Fig. 3B and Supporting Information Movie S2A/B), it was clear that overexpression of *whiB* and deletion of *bldO* had the same dramatic effect on the extent and timing of sporulation septation.

We previously showed that WhiA and WhiB function cooperatively to co‐control expression of a common set of WhiAB target genes (Bush *et al*., 2016). Therefore, in parallel we additionally assessed the effect of expressing *whiA* constitutively using the ermE* promoter in the wild‐type background. In contrast to *whiB*, expressing *whiA* from the ermE* promoter had no effect on the timing or extent of development (Supporting Information Movie S7A/B).

## Discussion

**Figure 9:**
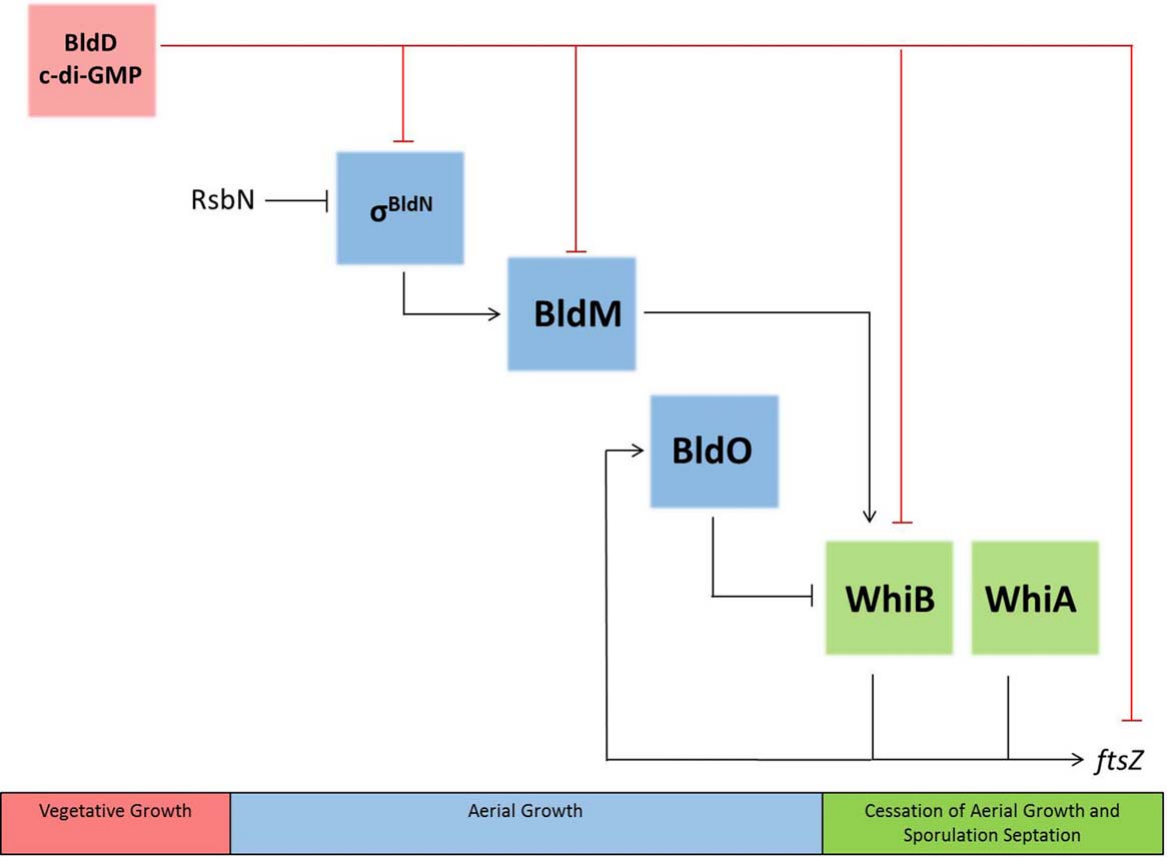
Simplified representation of the developmental regulatory network, highlighting the involvement of BldO and the importance of *whiB* as a node (adapted from *Nat Rev Microbiol* 2015, **13**, 749–760, with permission). Both BldM (Al‐Bassam *et al*., 2014) and σ^BldN^ (of which RsbN is the cognate anti‐sigma factor; Bibb *et al*., 2012) are required for the formation of aerial hyphae. During vegetative growth, *whiB* expression is repressed both by BldD‐(c‐di‐GMP) and by BldO. Relief from repression by BldD (presumably due to a drop in c‐di‐GMP levels) and BldO (by an unknown mechanism) and activation by BldM leads to expression of *whiB*. Once produced, WhiB combines with WhiA to initiate developmental cell division.

This work identifies BldO as a second repressor of *whiB* expression, in addition to BldD‐(c‐di‐GMP) (Fig. 9). As the master regulator that sits at the top of the regulatory cascade, BldD‐(c‐di‐GMP) represses expression of a large regulon of ∼170 sporulation genes during vegetative growth. This regulon not only includes almost all the genes of the core transcriptional regulatory cascade itself (with the notable exception of *whiA*; see below) but also includes genes encoding crucial components of the cell division and chromosome segregation machineries required for sporulation septation (Fig. 9) (Elliot *et al*., 2001; den Hengst *et al*., 2010; Tschowri *et al*., 2014; Bush *et al*., 2015). Release of BldD‐(c‐di‐GMP)‐mediated repression, presumably caused by a drop in c‐di‐GMP levels, triggers entry into development (Tschowri *et al*., 2014). BldO functions as a second repressor of *whiB*, but unlike BldD, BldO appears to regulate *whiB* as its only target. Because WhiB plays a key role in arresting hyphal growth and initiating developmental cell division, loss of either BldD or BldO is sufficient to promote precocious hypersporulation. Thus, together, the inhibitory activities of BldD and BldO help sustain a period of vegetative growth prior to hyphal differentiation into spores.

This work further highlights *whiB* as a central node in the developmental regulatory cascade, subject to BldO‐ and BldD‐mediated repression (den Hengst *et al*., 2010; Bush *et al*., 2015), and to activation by BldM (Al‐Bassam *et al*., 2014) (Fig. 9). WhiA and WhiB act together to co‐control the same set of promoters to initiate developmental cell division in *Streptomyces* (Bush *et al*., 2016). With the exception of WhiA autoregulation (Bush *et al*., 2013), no direct regulator of *whiA* expression has been identified. Instead, WhiA is constitutively present throughout the *Streptomyces* life cycle, but it only binds to its target promoters at the onset of sporulation (Bush *et al*., 2013). This is because WhiA and WhiB function cooperatively and *in vivo* DNA binding by WhiA depends on WhiB (and *vice versa*) (Bush *et al*., 2016). As a consequence, the regulation of *whiB* expression is key in controlling the switch between hyphal growth and sporulation.

We originally identified *sven0965* (now *bldO*) as an interesting candidate for mutagenesis because it is directly activated by WhiAB (Bush *et al*., 2016). Thus, early in development BldO represses *whiB*, and when BldO repression is somehow relieved, WhiB combines with WhiA to stimulate further expression of *bldO* (Fig. 9). It seems likely that this arrangement results in a transient window of WhiB expression in sporogenic hyphae. The potential significance of this feedback loop will be the subject of future investigation. How might BldO repression of *whiB* be relieved? BldO is undetectable in fully differentiated cultures (Fig. 4), suggesting that it is actively degraded during sporulation. However, BldO is also a member of the MerR family of transcription factors (Supporting Information Fig. S4). MerR family members consist of an N‐terminal DNA‐binding domain containing a winged helix‐turn‐helix motif, a C‐terminal effector‐recognition domain and an interconnecting linker region that consists of a long α‐helix that interacts with the same helix in the other subunit, forming a coiled‐coil responsible for dimerization (Brown *et al*., 2003; Hobman *et al*., 2005). MerR family proteins share similarity only within their DNA‐binding domains; as different family members bind unrelated effectors, their C‐terminal domains are variable and show little, if any, similarity to one another (Supporting Information Fig. S4) (Brown *et al*., 2003; Hobman *et al*., 2005). This raises the speculative possibility that BldO activity might be controlled by a ligand that interacts with its C‐terminal domain.

## Experimental procedures

### Bacterial strains, plasmids, oligonucleotides and growth media

Strains, plasmids and oligonucleotides used in this study are described in Supporting Information Table S1. *Escherichia coli* K‐12 strain DH5α was used for plasmid and cosmid propagation. BW25113 (Datsenko and Wanner, 2000) containing a λ RED plasmid, pIJ790, was used to create disrupted cosmids. Cosmids and plasmids were conjugated from the *dam dcm hsdS E. coli* strain ET12567 containing pUZ8002 (Paget *et al*., 1999) as described previously (Kieser *et al*., 2000; Gust *et al*., 2003, 2004). Phenotypic analysis of *S. venezuelae* and derived strains was conducted on solid media (Difco™ Nutrient Agar [DNA]) or in liquid culture (Difco™ Nutrient Broth [DNB].

### Construction and complementation of an *S. venezuelae bldO* null mutant

Using ‘Redirect’ PCR targeting (Gust *et al*., 2003; 2004), *bldO* mutants were generated in which the central (564bp) coding region was replaced with a single apramycin resistance (*apr*) cassette between the first and last 12 codons. A cosmid library that covers > 98% of the *S. venezuelae* genome (M.J. Bibb and M.J. Buttner, unpublished) is fully documented at http://strepdb.streptomyces.org.uk/. Cosmid Sv‐4‐G11 was introduced into *E. coli* BW25113 containing pIJ790 and the *bldO* gene (*sven0965*) was replaced with the *apr‐oriT* cassette amplified from pIJ773 using the primer pairs bldOdis_F and bldOdis_R. The resulting disrupted cosmids were confirmed by restriction digestion and by PCR analysis using the flanking primers bldOcon_F and bldOcon_R, and introduced into *S. venezuelae* by conjugation (Kieser *et al*., 2000). Null mutant derivatives, generated by double crossing over, were identified by their apramycin‐resistant, kanamycin‐sensitive and morphological phenotypes, and their chromosomal structures were confirmed by PCR analysis using the flanking primers bldOcon_F and bldOcon_R. A representative *bldO* null mutant was designated SV52. For complementation, *bldO* was amplified with the primers bldOcomp_F and bldOcomp_R, generating an 849bp fragment carrying the coding sequence and the *bldO* promoter, and cloned into HindIII‐KpnI/Asp718 cut pMS82 (Gregory *et al*., 2003) to create pIJ10612. The plasmid was introduced into the *bldO* mutant by conjugation and fully complemented all aspects of the mutant phenotype.

### Construction of an *S. venezuelae bldO whiB* double mutant

To make a *bldO whiB* double mutant, a cosmid was first made in which the central coding region of *whiB* was replaced with a single hygromycin (*hyg*) cassette between the start and stop codons. Cosmid PL2‐E20 was introduced into *E. coli* BW25113 containing pIJ790 and the *whiB* gene (*sven2776*) was replaced with the *hyg‐oriT* cassette amplified from pIJ10700 using the primer pairs whiBdis_F and whiBdis_R. The resulting disrupted cosmid was confirmed by restriction digestion and by PCR analysis using the flanking primers whiBcon_F and whiBcon_R, and introduced into WT *S. venezuelae* and SV52 (*bldO::apr*). Null mutant derivatives, generated by double crossing over, were identified by their hygromycin‐resistant, kanamycin‐sensitive and morphological phenotypes, and their chromosomal structures were confirmed by PCR analysis using the flanking primers whiBcon_F and whiBcon_R. A representative *ΔwhiB::hyg* mutant was designated SV53 and had an identical phenotype to the *ΔwhiB::apr* mutant SV7 (Bush *et al*., 2016). For complementation of SV53, plasmid pIJ10617 was created, in which the *whiB* promoter and coding region was (XbaI/EcoRV) cloned into the apramycin‐marked pSET152 using the whiBcomp_F/R primers (Supporting Information Table S1). pIJ10617 fully complemented SV53. A representative *ΔbldO::apr ΔwhiB::hyg* mutant was designated SV54.

### Construction of 3x‐FLAG BldO complemented *S. venezuelae* strains

To engineer an *S. venezuelae* strain expressing a form of BldO with either an N‐terminal or C‐terminal, triple‐FLAG tag (DYKDHDGDYKDHDIDYKDDDDK), pMS82‐derived constructs, pIJ10613 and pIJ10614, were created via a two‐step fusion‐PCR approach. For both constructs, in the first step, the Sv‐4‐G11 cosmid was used as a template for two separate PCR‐reactions. For the N‐terminal tag, the first reaction amplified the promoter region of the *whiB* gene using the primer pair bldONFLAG_P1 and bldONFLAG_P2. The second reaction amplified the coding region of the *whiB* gene using the primer pair bldONFLAG_P3 and bldONFLAG_P4. Together the bldONFLAG_P2 and bldONFLAG_P3 primers contain the sequence encoding the triple‐FLAG tag via a 24bp overlapping section. In the second step, a PCR reaction using the primers bldONFLAG_P1 and bldONFLAG_P4 was used to amplify the entire *whiB* gene and its promoter, fusing the two products from step 1 together and incorporating the 3xFLAG tag sequence between them. For the C‐terminal tag, in the first step, the first reaction amplified the promoter and coding region of the *whiB* gene using the primer pair bldOCFLAG_P1 and bldOCFLAG_P2. The second reaction amplified a short section of sequence following the coding region of the *whiB* gene but including the *whiB* stop codon using the primer pair bldOCFLAG_P3 and bldOCFLAG_P4. Together the bldOCFLAG_P2 and bldOCFLAG_P3 primers contain the sequence encoding the triple‐FLAG tag via a 24bp overlapping section. In the second step, a PCR reaction using the primers bldOCFLAG_P1 and bldOCFLAG_P4 was used to amplify the entire *whiB* gene and its promoter, fusing the two products from step 1 together and incorporating the 3xFLAG tag sequence between them. Both (NFLAG and CFLAG) sets of P1 and P4 primers additionally contain the HindIII and KpnI sites respectively to enable cloning into HindIII, KpnI‐cut pMS82. The resulting vectors were named pIJ10613 and pIJ10614 respectively. Both plasmids were introduced into the *ΔbldO::apr* mutant SV52 by conjugation and their ability to restore aerial hyphae formation and normal sporulation was assessed both on solid DNA and in liquid DNB medium. The resulting SV52‐pIJ10614 strain was used in the ChIP‐seq experiment described in this study. Genes expressed from pMS82‐derived constructs can be subject to overexpression due to the presence of a promoter from the apramycin resistance gene, upstream of the MCS. Therefore, to accurately assess BldO‐levels in Western blots, a derivative of pMS82, containing an extended MCS (pIJ10750) was first modified to remove this promoter. For this, PCR was conducted using the 82‐apr_1 and 82‐apr_2 primers and pIJ10750 as a template. After cutting with HindIII, the plasmid was re‐ligated to create pIJ10770. The *bldO‐3xFLAG* was subsequently sub‐cloned from pIJ10614 using the HindIII and KpnI restriction enzymes to create pIJ10616. pIJ10616 was introduced into the *ΔbldO::apr* mutant SV52 and fully complemented all aspects of the mutant phenotype. The resulting SV52‐pIJ10616 strain was used in Western blotting analysis to assess BldO levels throughout development.

### Construction of *bldOp‐ypet* transcriptional fusion

To construct a *bldOp‐ypet* fusion, the *bldO*Ypet_F and *bldO*Ypet_R primers were used and the 216bp product cloned into HindIII/XhoI‐cut pIJ10773 to create the pIJ10615 plasmid. This plasmid includes a *bldOp‐ypet* fusion in which the six nucleotides GGCGTG that normally precede the *bldO* start codon are replaced by the CTCGAG sequence, encoding the XhoI site, ensuring that the *bldO* Shine‐Dalgarno sequence remains intact and appropriately positioned relative to the start codon. pIJ10615 and pIJ10773 (empty vector control) were conjugated into the фBT1 chromosomal attachment site of wild‐type *S. venezuelae* for subsequent flourescence microscopy.

### Time‐lapse imaging of *S. venezuelae*

Fluorescent time‐lapse imaging was conducted essentially as described previously (Schlimpert *et al*., 2016). Before imaging, fresh *S. venezuelae* spores for each of the strains imaged were first prepared by inoculating 30 ml cultures of DNB with 10 µl of the appropriate spore stock. Cells were cultured at 30°C and 250 r.p.m. until fully differentiated (16–24 h for hypersporulating strains, otherwise 36–40 h). One millilitre of each culture was spun down at 400 xg for 1 min to pellet any remaining mycelium, the supernatant diluted 1:20 in DNB and 40 µl transferred to the cell loading well of a prepared B04A microfluidic plate (Merck‐Millipore). The ONIX manifold was then sealed to the B04A plate before transferring to the environmental chamber, pre‐incubated at 30°C. Spores were loaded onto the B04A plate, at 4 psi for 15 sec using the ONIX microfluidic perfusion system. Fresh DNB medium was set to flow at 6 psi during the entirety of the imaging experiment. The system was left to equilibrate for 2 h prior to imaging.

Imaging was conducted using a Zeiss Axio Observer.Z1 widefield microscope equipped with a sCMOS camera (Hamamatsu Orca FLASH 4), a metal‐halide lamp (HXP 120V), a hardware autofocus (Definitive Focus), a 96‐well stage insert, an environmental chamber, a 100x 1.46 NA Oil DIC objective and the Zeiss 46 HE shift free (excitation500/25 nm, emission 535/30 nm) filter set. For imaging of *ftsZ‐ypet* strains, DIC images were captured with a 150 ms exposure time, YFP images were captured with a 350 ms exposure time. Images were taken every 20 min for hypersporulating strains (the *bldO* mutant and the WhiB overexpression strain), every hour (for wild type *S. venezuelae*), or every hour for the first 8 h and every 20 min subsequently (for the complemented *bldO* mutant and the WhiA overexpression strain). For imaging of the *bldOp‐ypet* transcriptional fusion strain, DIC images were captured with a 150 ms exposure time and YFP images were captured with a 250 ms exposure time. Images were taken every hour. In all experiments, multiple *x*/*y* positions were imaged for each strain and in each experiment. Representative images were transferred to the Fiji software package (http://fiji.sc/Fiji), manipulated and converted into the movie files presented here, as described previously (Schlimpert *et al*., 2016).

### Microscopy of *S. venezuelae* grown in liquid cultures

To assess the phenotype in liquid culture of various strains used in this study, cells were grown in 30 ml DNB cultures at 30°C, 250 r.p.m. in flasks with springs to assist aeration. After 16 h, 2 µl of culture was removed and added to the surface of a 1% agarose pad, a coverslip added, and imaged using the 100x DIC Objective of the Leica DM 6000 microscope with Leica DFC360 FX (cooled monochrome) camera. Images were manipulated using the Fiji software package (http://fiji.sc/Fiji). Prior to assaying BldO‐3xFLAG levels, cultures were grown and prepared in the same way but images captured using a Zeiss Axio Observer.Z1 widefield microscope.

For fluorescence microscopy of the *bldOp‐ypet* strain (and the strain carrying the empty vector), imaging was conducted using a Zeiss Axio Observer.Z1 widefield microscope. FM® 4‐64 Dye (*N*‐(3‐Triethylammoniumpropyl)−4‐(6‐(4‐(Diethylamino) Phenyl) Hexatrienyl) Pyridinium Dibromide) (Molecular probes; final concentration 5 µg/ml) was added to samples prior to imaging. DIC images were captured with a 150 ms exposure time, YFP images were captured with a 1000 ms exposure time and RFP images captured with a 250 ms exposure time.

### For Chromatin immunoprecipitation, library construction, sequencing, ChIP‐seq data analysis, qRT‐PCR, DNase I footprinting, Western blotting, SEM

Please see Text S1 in the Supporting Information.

## Supporting information

Supporting Information

Supporting Information

Supporting Information

Supporting Information

Supporting Information

Supporting Information

Supporting Information

Supporting Information

Supporting Information

Supporting Information

Supporting Information

Supporting Information

Supporting Information

Supporting Information

Supporting Information
