# Supplementary material for: Multi‐layered inhibition of Streptomyces development: BldO is a dedicated repressor of whiB
Source: Mol Microbiol. 2017 Mar 27;104(5):700–11. doi: 10.1111/mmi.13663 (PMC5485038; doi:10.1111/mmi.13663)
Supplement: Supplementary file 15 — Supporting Information [file MMI-104-700-s015.pdf]

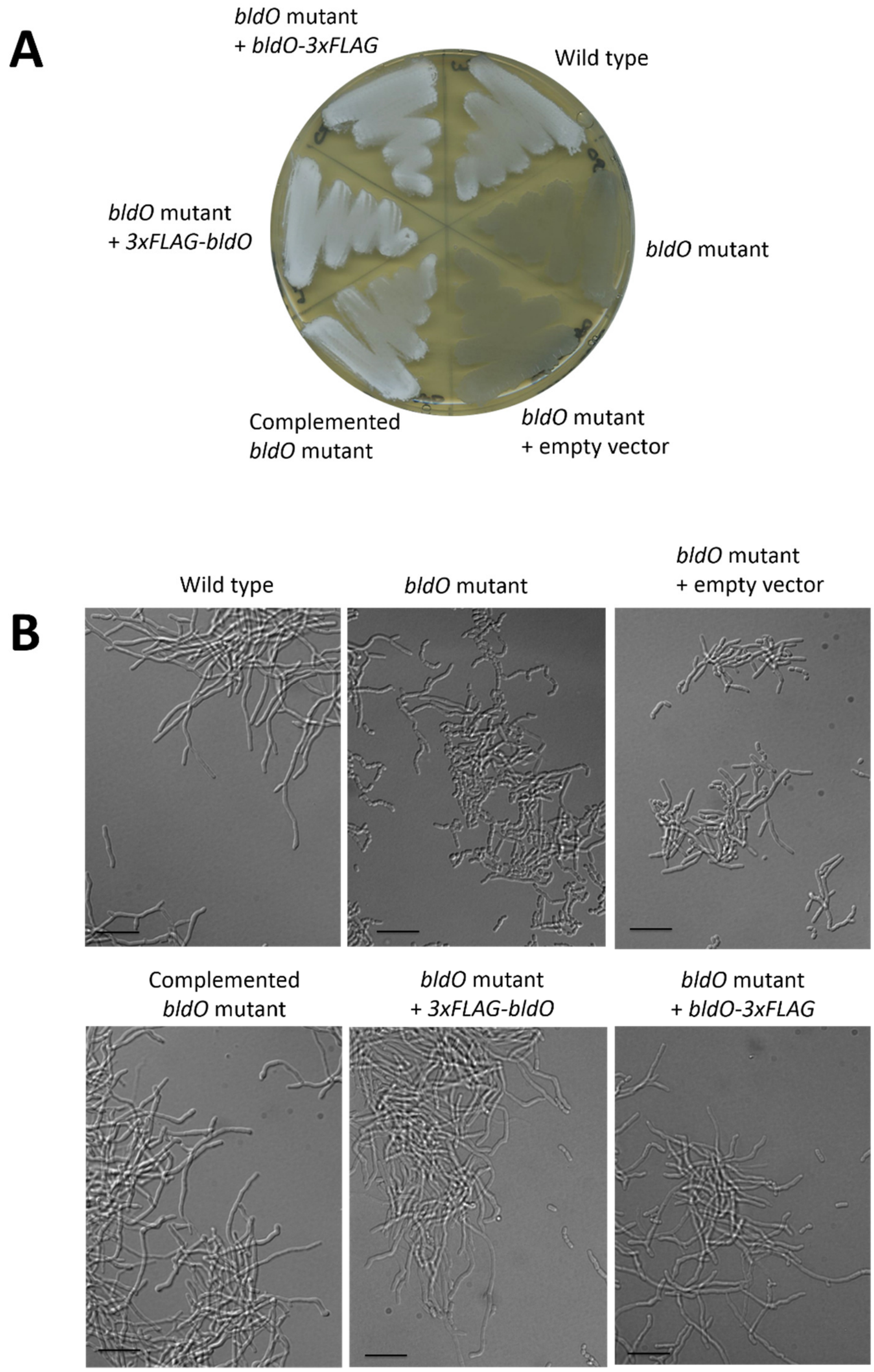

FIG S1. Both the N-terminal and C-terminal 3xFLAG tagged versions of BldO are functional. Shown are the phenotypes of wild-type *S.venezuelae*, the *bldO* mutant, the *bldO* mutant carrying the empty vector, the complemented *bldO* mutant, the *bldO* mutant complemented with N-terminally 3xFLAG-tagged *bldO* and the *bldO* mutant complemented with C-terminally 3xFLAG-tagged *bldO*, either on solid medium (A) or in liquid culture (B). Strains were photographed after four days of growth on solid DNA medium and DIC images taken after 16 hours' growth in liquid DNB medium. Scale bars = 10 µm.

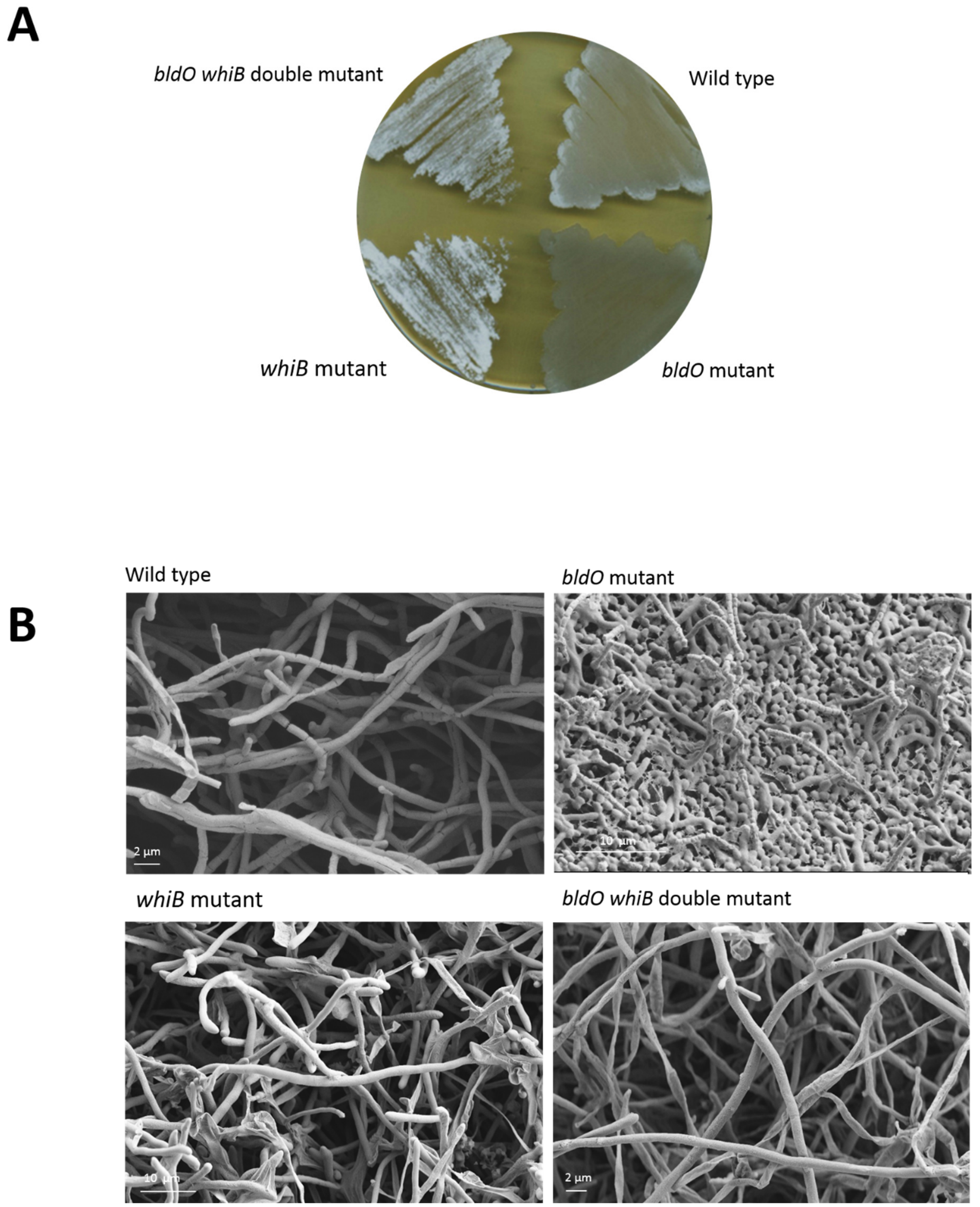

FIG S2. A *bldO whiB* double mutant has the same phenotype as the *whiB* mutant. Photograph (A) and scanning electron micrographs (B) showing the phenotypes of wild-type *S.venezuelae*, the *bldO* mutant, the *whiB* mutant, and the *bldO whiB* double mutant, after four days of growth on DNB medium.

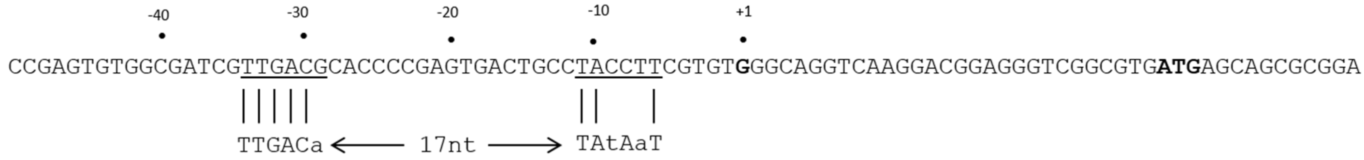

FIG S3. The *bldO* transcriptional start site lies 29 nucleotides upstream of the annotated start codon. The position of the transcriptional start-site, as defined by 5'RACE is indicated relative to the predicted -10/-35 promoter elements. The -10 and -35 consensus sequences for σ^{HrdB}-dependent promoters are also shown for comparison.

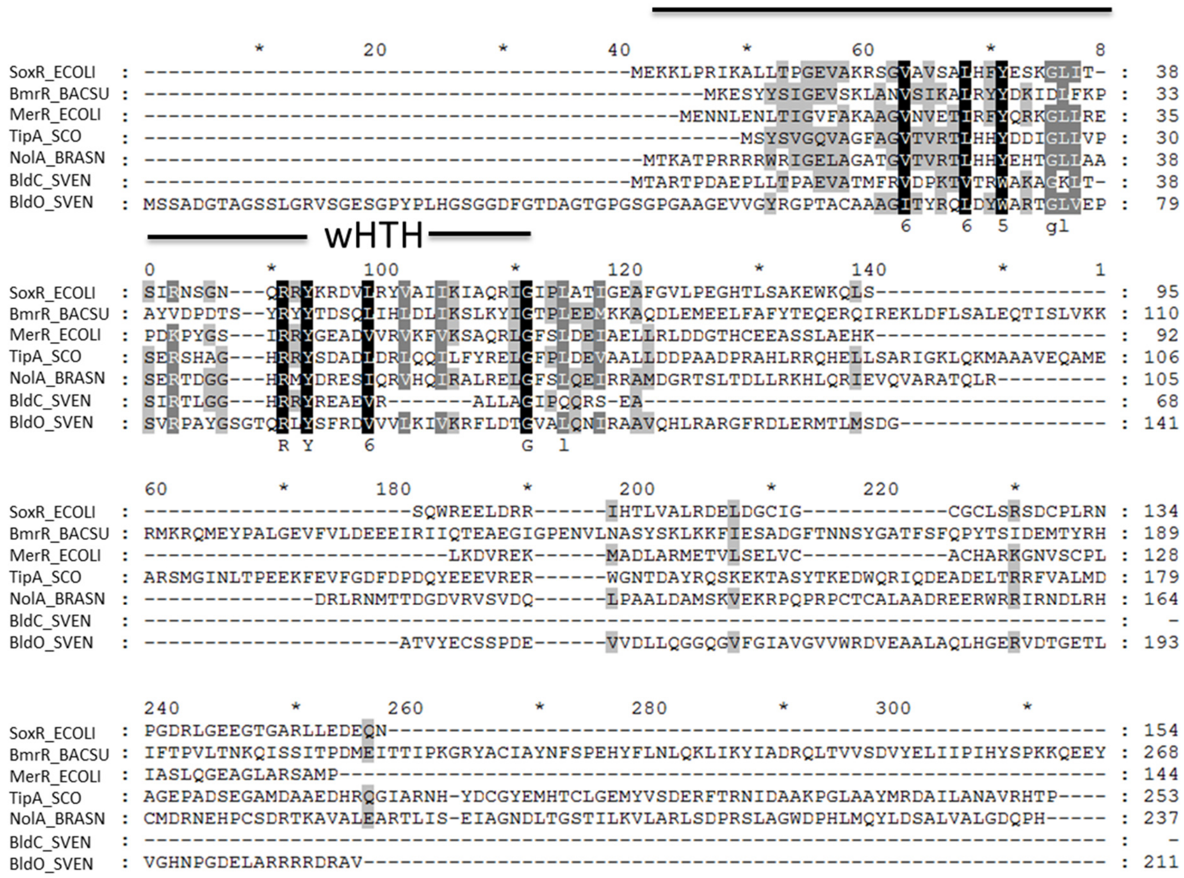

FIG S4. BldO is a member of the MerR-family proteins, as shown by protein sequence alignment. Sequences shown were derived from the following entries in the UniProtKB database (http://www.uniprot.org/uniprot/): BldO_SVEN (*Streptomyces venezuelae*; F2RBC4); BldC_SVEN (*Streptomyces venezuelae*; F2REK9); SoxR_ECOLI (*Escherichia coli;* P0ACS2);BmrR_BACSU (*Bacillus subtilis;* P39075); MerR_ECOLI (*Escherichia coli;* Q7BT49); TipA_SCO (*Streptomyces coelicolor*; P0A4T8); NolA_BRASN (*Bradyrhizobium sp. strain NC92;* P50330). Alignment was made using ClustalOmega (https://www.ebi.ac.uk/Tools/msa/clustalo/) and viewed in the GeneDoc software.

Movie S1 Time-lapse microscopy of the wild type strain carrying the FtsZ-YPet fusion. DIC (A) and YFP-channel (B) movies are at 5 frames per second. The time following the first image is indicated at the bottom left. Images were taken every 1 hour (DIC 150 ms; YFP 350 ms). Movies were assembled in the Fiji software package (http://fiji.sc/Fiji).

Movie S2 Time-lapse microscopy of the *bldO* mutant strain carrying the FtsZ-YPet fusion. DIC (A) and YFP-channel (B) movies are at 5 frames per second. The time following the first image is indicated at the top left. Images were taken every 20 mins (DIC 150 ms; YFP 350 ms). Movies were assembled in the Fiji software package (http://fiji.sc/Fiji).

Movie S3 Time-lapse microscopy of the complemented *bldO* mutant strain carrying the FtsZ-YPet fusion. DIC (A) and YFP-channel (B) movies are at 5 frames per second. The time following the first image is indicated at the bottom left. Images were taken every 1 hour for first 8 hours and every 20 mins subsequently (DIC 150 ms; YFP 350 ms). Movies were assembled in the Fiji software package (http://fiji.sc/Fiji).

Movie S4 Time-lapse microscopy of the wild type strain expressing the *bldOp-ypet* fusion. DIC (A) and YFP-channel (B) movies are at 5 frames per second. The time following the first image is indicated at the bottom left. Images were taken every 1 hour (DIC 150 ms; YFP 250ms). Movies were assembled in the Fiji software package (http://fiji.sc/Fiji).

Movie S5 Time-lapse microscopy of the wild type strain carrying the empty vector (*ypet* without promoter). DIC (A) and YFP-channel (B) movies are at 5 frames per second. The time following the first image is indicated at the bottom left. Images were taken every 1 hour (DIC 150 ms; YFP 250ms). Movies were assembled in the Fiji software package (http://fiji.sc/Fiji).

Movie S6 Time-lapse microscopy of the wild-type strain constitutively expressing *whiB* from the *ermE** promoter and carrying the FtsZ-YPet fusion. DIC (A) and YFP-channel (B) movies are at at 5 frames per second. The time following the first image is indicated at the bottom left. Images were taken every 1 hour for first 8 hours and every 20 mins subsequently (DIC 150 ms; YFP 350 ms). Movies were assembled in the Fiji software package (http://fiji.sc/Fiji).

Movie S7 Time-lapse microscopy of the wild-type strain constitutively expressing *whiA* from the *ermE** promoter and carrying the FtsZ-YPet fusion. DIC (A) and YFP-channel (B) movies are at at 5 frames per second. The time following the first image is indicated at the bottom left. Images were taken every 1 hour for first 8 hours and every 20 mins subsequently (DIC 150 ms; YFP 350 ms). Movies were assembled in the Fiji software package (http://fiji.sc/Fiji).

Text S1 – Supplementary Experimental Procedures

Chromatin immunoprecipitation, library construction, sequencing and ChIP-seq data analysis. ChIP was conducted using M2 gel suspension (Sigma-Aldrich A2220) as described previously (Bush *et al.*, 2013) for the strains *S. venezuelae* ATCC 10712 and SV52-pIJ10613 (*ΔbldO::apr attB$_{ΦBT1}$::bldO-3xFLAG* – Table S1) except that cultures were grown in DNB liquid medium. Library construction and sequencing were performed as described previously (Bush *et al.*, 2013) by The Earlham Institute, Norwich Research Park Norwich, UK. ChIP-seq data analysis was conducted as described previously (Bush *et al.*, 2013), except that here for both the "diff" and "apv" values the corresponding value for the wild-type (WT) negative control was subtracted from the initial value to arrive at a final value.

qRT-PCR

Mycelial pellets from DNB cultures were washed in PBS and resuspended in 900 µl lysis solution (400 µl phenol [pH4.3], 100 µl chlorophorm:isoamyl alcohol (24 : 1) and 400 µl RLT buffer [Qiagen]) with lysing matrix B (MP Biomedicals) and homogenised using a FastPrep FP120 Cell Disruptor (Thermo Savant). Two pulses of 30 s of intensity 6.0 were applied with cooling down for 1 min on ice between pulses. Supernatants were centrifuged for 15 min, full-speed on a bench-top centrifuge at 4°C and then treated according to the instructions given in the RNEasy Kit (Qiagen). The RNA samples were treated with on-column DNase I (Qiagen), followed by an additional DNase I treatment (Turbo DNA-free, Ambion) until they were free of DNA contamination (determined by PCR amplification of *hrdB*). RNA was quantified and equal amounts (500 ng) of total RNA from each sample was converted to cDNA using SuperScript II reverse transcriptase and random primers (Invitrogen). cDNA was then used as template in qRT-PCR performed using the SensiFAST SYBR No-ROX kit (Bioline). Three technical replicates were used for each gene. Specific qPCR primers (Table S1, final concentration of 250 nM) were used to amplify the *whiB* target gene (whiBqRT_F and whiBqRT_R) and the *hrdB* reference gene (hrdBqRT_F and hrdBqRT_R). To normalize for differing primer efficiency, a standard curve was constructed using chromosomal DNA. Melting curve analysis was used to confirm the production of a specific single product from each primer pair. qRT-PCR was performed using a CFX96 Touch instrument using hardshell white PCR plates (BioRad), sealed with thermostable film covers (Thermo). PCR products were detected with SYBR green fluorescent dye and amplified according to the following protocol: 95°C, 3 min, then 45 cycles at 95°C 5 sec, 60°C 10 sec and 72°C 7 sec. Melting curves

were generated at 65 to 95°C with 0.5°C increments. The experiments (including RNA extraction) was repeated once independently. The BioRad CFX manager software was used to calculate starting quantity (SQ) values for *whiB* at each time point. These values were divided by the mean SQ value derived from the *hrdB* reference at the corresponding time points, generating a value for relative expression. The resulting values were normalised against the mean relative expression of the wild type at 10 hours, which was set to 1. The resulting normalised relative expression is reported in Figure 8.

DNase I Footprinting. DNase I footprinting experiments to study BldO binding to the *whiB* promoter were carried out essentially as described previously (Bush *et al.*, 2103) and according to the description supplied with the Sure Track footprinting kit (Amersham Pharmacia Biotech). DNA probes containing the *whiB2p* promoter were prepared by PCR using the primers whiB2p_F and whiB2p_R (Table S1). To study binding of BldO to the forward strand, whiB2p_F was first radiolabelled and to study binding to the reverse strand, whiB2p_R was first radiolabelled.

Western Blotting. Samples of frozen mycelium, originating from 5 ml liquid DNB cultures, were resuspended in 0.4 ml ice-cold sonication buffer [20 mM Tris pH 8.0, 5 mM EDTA, 1 x EDTA-free protease inhibitors (Roche)] and sonicated (5x 15 sec on/15 sec off) at 4.5 micron amplitude. Lysates were then centrifuged at 16,000 xg for 15 min at 4˚C to remove cell debris. Total protein concentration was determined using the Bradford assay (Biorad). 2.5 µg of total protein from each time point was loaded in triplicate into a microplate (proteinsimple #043-165) and anti-FLAG antibody (Sigma F4725) diluted 1:100. BldO-3xFLAG levels, originating from the SV52-pIJ10616 strain and the wild type negative control (Table S1) were then assayed using the automated Western blotting machine Wes (ProteinSimple, San Jose, CA), according to the manufacturer's guidelines.

Scanning electron microscopy. Colonies were mounted on the surface of an aluminum stub with optimal cutting temperature compound (Agar Scientific Ltd, Essex, UK), plunged into liquid nitrogen slush at approximately -210°C to cryopreserve the material, and transferred to the cryostage of an Alto 2500 cryotransfer system (Gatan, Oxford, England) attached to a Zeiss Supra 55 VP field emission gun scanning electron microscope (Carl Zeiss Ltd, Germany). The surface frost was sublimated at -95°C for 3 min before the sample was sputter coated with platinum for 2 min at 10 mA at below -110°C. Finally, the sample was

moved onto the cryostage in the main chamber of the microscope, held at approximately -130°C, and viewed at 1.2 to 5.0 kV.

References

Bush, M.J., Bibb, M.J., Chandra, G., Findlay, K.C., Buttner, M.J. (2013) Genes required for aerial growth, cell division, and chromosome segregation are targets of WhiA before sporulation in *Streptomyces venezuelae*. *mBio* **4:** e00684-13.

Table S1 Strains, Plasmids and Oligonucleotide primers used in this study

**Strains**	**Relevant genotype/comments**	**Source/reference**
S.venezuelae		
ATCC10712	Wild type	
SV7	Δ*whiB::apr*	Bush et al., (2016)
SV52	Δ*bldO::apr*	This work
SV53	Δ*whiB::hyg*	This work
SV53-pIJ10617	Δ*whiB::hyg* with pIJ10617 integrated at the ΦC31 attachment site	This work
SV54	*Sven*Δ*bldO::apr Sven*Δ*whiB::hyg*	This work
SV52-pMS82	Δ*bldO::apr* with pMS82 integrated at the ΦBT1 attachment site	This work
SV52-pIJ10612	Δ*bldO::apr* with pIJ10612 integrated at the ΦBT1 attachment site	This work
Sven-pIJ10753	Wild type with pIJ10753 integrated at the ΦBT1 attachment site	This work
SV52-pIJ10753	Δ*bldO::apr* with pIJ10753 integrated at the ΦBT1 attachment site	This work
SV52-pIJ10612-pKF351	Δ*bldO::apr* with pIJ10612 integrated at the ΦBT1 attachment site and pKF351 integrated at the ΦC31 attachment site	This work
SV52-pIJ10613	Δ*bldO::apr* with pIJ10613 integrated at the ΦBT1 attachment site	This work
SV52-pIJ10614	Δ*bldO::apr* with pIJ10614 integrated at the ΦBT1 attachment site	This work
Sven-pIJ10605-pKF351	Wild type with pIJ10605 integrated at the ΦBT1 attachment site and pKF351 integrated at the ΦC31 attachment site	This work
Sven-pIJ10606-pKF351	Wild type with pIJ10606 integrated at the ΦBT1 attachment site and pKF351 integrated at the ΦC31 attachment site	This work
Sven-pIJ10615	Wild type with pIJ10615 integrated at the ΦBT1 attachment site	This work
Sven-pIJ10773	Wild type with pIJ10773 integrated at the ΦBT1 attachment site	This work
SV52-pIJ10616	Δ*bldO::apr* with pIJ10616 integrated at the ΦBT1 attachment site	This work
E.coli		
ET12567(pUZ8002)	ET12567 containing helper plasmid pUZ8002	Paget et al., (1999)
BW25113	Δ(*araD-araB*)*567* Δ *lacZ4787*(::*rrnB-4*) *lacIp-4000*(*lacI*^{Q}), l-*rpoS369*(*Am*) *rph-1* Δ(*rhaD-rhaB*)*568 hsdR514*	Datsenko and Wanner (2000)
**Plasmids**		
pIJ773	Plasmid template for amplification of the *apr oriT* cassette for 'Redirect' PCR-targeting	Gust et al., (2003)
pIJ10700	Plasmid template for amplification of the *hyg oriT* cassette for 'Redirect' PCR-targeting	Gust et al., (2003)
pIJ790	Modified l RED recombination plasmid [*oriR101*] [*repA101*(ts)] *araBp-gam-be-exo*	Gust et al., (2003)
pMS82	Plasmid cloning vector for the conjugal transfer of DNA from *E. coli* to *Streptomyces* spp. Integrates site specifically at the ΦBT1 attachment site (Hyg^R)	Gregory et al., (2003)

pIJ10257	Plasmid cloning vector for the conjugal transfer of DNA (under control of the ermE* constitutive promoter) from *E. coli* to *Streptomyces* spp. Integrates site specifically at the ΦBT1 attachment site (Hyg^R).	Hong et al., (2005)
pSET152	Plasmid cloning vector for the conjugal transfer of DNA from *E. coli* to *Streptomyces* spp. Integrates site specifically at the ΦC31 attachment site (Apr^R)	Combes et al., (2002)
pIJ10612	pMS82 carrying *bldO* driven from its own promoter	This work
pIJ10613	pMS82 carrying sequence encoding 3xFLAG-BldO driven from *bldO* promoter	This work
pIJ10614	pMS82 carrying sequence encoding BldO-3xFLAG driven from *bldO* promoter	This work
pIJ10753	pMS82 carrying *ftsZ-ypet* driven from the *ftsZ* promoter	S.Schlimpert (unpublished)
pIJ10605	pIJ10257 carrying *whiA*, driven by ermE*	Bush et al., (2016)
pIJ10606	pIJ10257 carrying *whiB*, driven by ermE*	Bush et al., (2016)
pKF351	pSET152 carrying *ftsZ-ypet* driven from the *ftsZ* promoter	Donczew et al., (2016)
pIJ10773	pMS82-based plasmid carrying the *ypet* gene for C-terminal transcriptional fusions. Integrates site specifically at the ΦBT1 attachment site (Hyg^R)	S.Schlimpert (unpublished)
pIJ10615	pIJ10773 with the *bldO* promoter cloned upstream of the *ypet* gene	This work
pIJ10750	pMS82 with an extended Multiple Cloning Site (MCS)	S.Schlimpert (unpublished)
pIJ10770	A derivative of pIJ10750 with the *apr* gene promoter deleted	S.Schlimpert (unpublished)
pIJ10616	pIJ10770 carrying a sequence encoding bldO-3xFLAG driven from its own promoter	This work
pIJ10617	pSET152 carrying *whiB* driven from its own promoter	This work

**Primers**	**Sequence**
bldOdis_F	gtgatgagcagcgcggacggtacggcagggagctcgctcattccggggatccgtcgacc
bldOdis_R	ctctcagacggccctgtcccgccggcgcctcgccagctctgtaggctggagctgcttc
bldOcon_F	ccgttccccggcgagaga
bldOcon_R	gctgcctcacaccactgac
bldOcomp_F	ggcgaagcttgggaccaacagtcagtgacg
bldOcomp_R	ggggtaccctctcagacggccctgtcc
whiBdis_F	caccacggggacgcacagaacagacgagggggcgcacatgattccggggatccgtcgacc
whiBdis_R	ggcctggcgcgcggggcccggcaggcttcgcgccgttcatgtaggctggagctgcttc
whiBcon_F	gtcgctggcatgccacca
whiBcon_R	gcgaccacgttctgcacg
whiBcomp_F	ctagtctagagccggtggcatgtgtcac
whiBcomp_R	cctccgatatcgcaggcttcgcgccgttc
bldONFLAG_P1	ggcgaagcttgggaccaacagtcagtgacg
bldONFLAG_P2	tcgatgtcgtggtccttgtagtcgccgtcgtggtccttgtagtccatcacgccgaccctccg
bldONFLAG_P3	cgactacaaggaccacgacatcgactacaaggacgatgacgacaagagcagcgcggacggtacg
bldONFLAG_P4	ggggtaccctctcagacggccctgtcc
bldOCFLAG_P1	ggcgaagcttgggaccaacagtcagtgacgccg
bldOCFLAG_P2	tcgatgtcgtggtccttgtagtcgccgtcgtggtccttgtagtcgacggccctgtcccgccg

bldOCFLAG_P3	cgactacaaggaccacgacatcgactacaaggacgatgacgacaagtgagagccgcctgaggggcc
bldOCFLAG_P4	ggggtaccgacaacgggcccgcagccg
whiB2p_F	gcagagtgaatacgaggccc
whiB2p_R	gccagccgagttcttcgtc
bldOYpet_F	ggcgaagcttgggaccaacagtcagtgacgcc
bldOYpet_R	ccgctcgaggaccctccgtccttgacctg
82-apr_1	tataaagcttagattctctcatatgatcatcg
82-apr_2	tataaagcttgataaacttatcatccccttttgctg
whiBqRT_F	gcgaggcgaaaaaggtctgc
whiBqRT_R	gagaggcctccccagattcc
hrdBqRT_F	tgttctgcgcagcctcaatc
hrdBqRT_R	ctcttcgctgcgacgctctt

References

1. **Bush, MJ, Chandra, G, Bibb, MJ, Findlay K., and Buttner, MJ**. 2016. Genome-wide chromatin immunoprecipitation sequencing analysis shows that WhiB is a transcription factor that co-controls its regulon with WhiA to initiate developmental cell division in *Streptomyces*. *mBio* **7:** e00523-16.
2. **Paget MSB, Chamberlin L, Atrih A, Foster SJ, Buttner MJ**. 1999. Evidence that the extracytoplasmic function sigma factor, σ^E, is required for normal cell wall structure in *Streptomyces coelicolor* A3(2). J. Bacteriol. **181:**204–211.
3. **Datsenko KA, Wanner BL**. 2000. One-step inactivation of chromosomal genes in *Escherichia coli* K-12 using PCR products. Proc. Natl. Acad. Sci. U. S. A. **97:**6640–6645.
4. **Gust B, Challis GL, Fowler K, Kieser T, Chater KF**. 2003. PCR-targeted *Streptomyces* gene replacement identifies a protein domain needed for biosynthesis of the sesquiterpene soil odor geosmin. Proc. Natl. Acad. Sci. U. S. A. **100:**1541–1546.
5. **Gregory MA, Till R, Smith MCM**. 2003. Integration site for *Streptomyces* phage ΦBT1 and development of site-specific integrating vectors. J. Bacteriol. **185:**5320–5323.
6. **Hong HJ, Hutchings MI, Hill LM, Buttner MJ**. 2005. The role of the novel Fem protein VanK in vancomycin resistance in Streptomyces coelicolor. J Biol Chem. **280**:13055-61.
7. **Combes P, Till R, Bee S, Smith MC.** 2002. The streptomyces genome contains multiple pseudo-attB sites for the (phi)C31-encoded site-specific recombination system. J Bacteriol. **184**:5746-52.
8. **Donczew M, Mackiewicz P, Wróbel A, Flärdh K, Zakrzewska-Czerwińska J, Jakimowicz D**. 2016. ParA and ParB coordinate chromosome segregation with cell elongation and division during Streptomyces sporulation. Open Biol. **6(4):**150263.
